# Four Weeks of Off-Season Training Improves Peak Oxygen Consumption in Female Field Hockey Players

**DOI:** 10.3390/sports5040089

**Published:** 2017-11-28

**Authors:** Lindsey T. Funch, Erik Lind, Larissa True, Deborah Van Langen, John T. Foley, James F. Hokanson

**Affiliations:** 1 Kinesiology Department, State University of New York at Cortland, P.O. Box 2000, Cortland, NY 13045, USA; lindsey.taylor02@cortland.edu (L.T.F.); erik.lind@cortland.edu (E.L.); larissa.true@cortland.edu (L.T.); deborah.vanlangen@cortland.edu (D.V.L.); 2 Physical Education Department, State University of New York at Cortland, P.O. Box 2000, Cortland, NY 13045, USA; john.foley@cortland.edu

**Keywords:** HIIT, tabata intervals, maximal oxygen consumption, intervals, non-traditional season training, field hockey, $\dot{V}O_{2\mathrm{peak}}$, HIT

## Abstract

The purpose of the study was to examine the changes in peak oxygen consumption ($\dot{V}O_{2\mathrm{peak}}$) and running economy (RE) following four-weeks of high intensity training and concurrent strength and conditioning during the off-season in collegiate female field hockey players. Fourteen female student-athletes (age 19.29 ± 0.91 years) were divided into two training groups, matched from baseline $\dot{V}O_{2\mathrm{peak}}$: High Intensity Training (HIT_run_; *n* = 8) and High Intensity Interval Training (HIIT; *n* = 6). Participants completed 12 training sessions. HIT_run_ consisted of 30 min of high-intensity running, while HIIT consisted of a series of whole-body high intensity Tabata-style intervals (75–85% of age predicted maximum heart rate) for a total of four minutes. In addition to the interval training, the off-season training included six resistance training sessions, three team practices, and concluded with a team scrimmage. $\dot{V}O_{2\mathrm{peak}}$ was measured pre- and post-training to determine the effectiveness of the training program. A two-way mixed (group × time) ANOVA showed a main effect of time with a statistically significant difference in $\dot{V}O_{2\mathrm{peak}}$ from pre- to post-testing, *F*(1, 12) = 12.657, *p* = 0.004, partial η^2^ = 0.041. Average (±SD) $\dot{V}O_{2\mathrm{peak}}$ increased from 44.64 ± 3.74 to 47.35 ± 3.16 mL·kg^−1^·min^−1^ for HIIT group and increased from 45.39 ± 2.80 to 48.22 ± 2.42 mL·kg^−1^·min^−1^ for HIT_run_ group. Given the similar improvement in aerobic power, coaches and training staff may find the time saving element of HIIT-type conditioning programs attractive.

## 1. Introduction

Athletes train year-round for competition, yet based on National Collegiate Athletic Association (NCAA) regulations collegiate athletes have limited organized practice times with coaches during non-competition seasons [1]. An important objective of off-season training is to improve overall fitness and prepare the athlete for the approaching competitive season [2]. With limited off-season organized practice time, it would be a benefit for both the coach and athlete to have the athlete arrive at the competition season with a high level of fitness.

A benefit of high intensity interval training (HIIT) is demonstrated improvement in $\dot{V}$O_2max_ accomplished with short amount of training time [3]. It has been well established that continuous endurance training increases maximal oxygen consumption so this type of sustained, steady-state exercise is a training method that has primarily been utilized for improving aerobic fitness [4,5]. However, traditional endurance training (e.g., steady-state running) typically requires a daily commitment of 30 min or more to obtain aerobic benefits [6,7,8]. Such a time commitment needed for cardiovascular training may reduce the amount of time or effectiveness that a coach and athlete can devote to other aspects of the training, such as game strategy and technical skill work.

HIIT is a method of training that consists of brief bouts of exercise at or near maximal effort, such as all-out sprints or cycling at a predetermined power output, interspersed with short recovery periods. The recovery or rest periods may be as short as 10 s to as long as four minutes. HIIT has been studied in sedentary and obese volunteers [9,10], and studied extensively in trained and moderately trained populations, and shown to improve aerobic capacity in these groups [11,12,13]. With training durations of as little as one or two minutes per session [3,13], HIIT requires a reduced time commitment when compared to traditional endurance training, while still producing cardiovascular benefits. Improved cardiovascular fitness brought about by high-intensity interval training has been demonstrated in the form of increased $\dot{V}$O_2max_, as well as improvements in cycling time trials and time to exhaustion tests that are associated with HIIT training [14]. 

HIIT could be used as a substitute for steady-state run training as a more time-efficient method of increasing or maintaining aerobic fitness for athletic teams and could allow for a greater amount of practice time to be made available for strength training, skill work, and game strategy. Many college athletic teams are allowed a short structured off-season practice schedule, as sanctioned by the NCAA. For fall sports, such as field hockey, the non-traditional segment, or out-of-season practices, can be held for five weeks in spring, with a maximum of 16 total practice days [1]. Aside from these few weeks of practice, the athletes are expected to maintain conditioning on their own or through outside training programs.

HIIT has been fairly widely studied as a method of improving the overall fitness of unfit and moderately fit populations [15,16]. Recently, Coakley and Passfield [17] reported greater increases in $\dot{V}$O_2max_ with four weeks of training with a mixed (moderate and high intensity) cycling training in untrained individuals, and Kohn et al. [18] reported the efficacy of HIIT in trained runners. Yet, research is limited regarding the aerobic benefits of HIIT programs in female athletic team populations. Few studies have examined the advantages of whole-body HIIT as compared with high-intensity steady state running or cycling in an all-female population. Thus, given the limited time that student athletes have for improving off-season cardiovascular endurance, and the lack of investigations that are specific to female participants, the purpose of this study was to examine the changes in peak oxygen consumption following a short four-week off-season training program. A second purpose was to compare the effectiveness of a HIIT training program with a more traditional steady-state run training (HIT_run_) on a population of athletic, college-age females during their off-season. It was hypothesized that: (A) Four weeks of off-season training would result in a significant increase in peak oxygen consumption in an athletic female population; and, (B) HIIT training would result in a significantly greater increase in $\dot{V}$O_2peak_ when compared to the steady-state run training, HIT_run_.

## 2. Methods

### 2.1. Participants

Fifteen female NCAA Division III field hockey team members volunteered to participate in a four-week, 12-session training program. Prior to preliminary testing and training, informed consent and the Physical Activity Readiness Questionnaire (PAR-Q) were completed by each participant following an explanation of all the procedures, benefits, and risks of the study. No individuals answered yes to any of the questions on the PAR-Q, and all of the volunteers were retained for the study. No attempt was made to control for phase of menstrual cycle of volunteers. All of the methods and procedures were approved by the researchers’ University Institutional Review Board prior to recruitment and data collection.

Following preliminary testing, the participants were matched and assigned to one of two training groups: a high intensity endurance (HIT_run_) training group or a whole-body high-intensity interval training group (HIIT). Due to injury, one participant was unable to complete the experimental training, and was therefore was not included in the final analysis (N = 14, aged 19.29 ± 0.91 years).

### 2.2. Procedures

**Figure 1:**
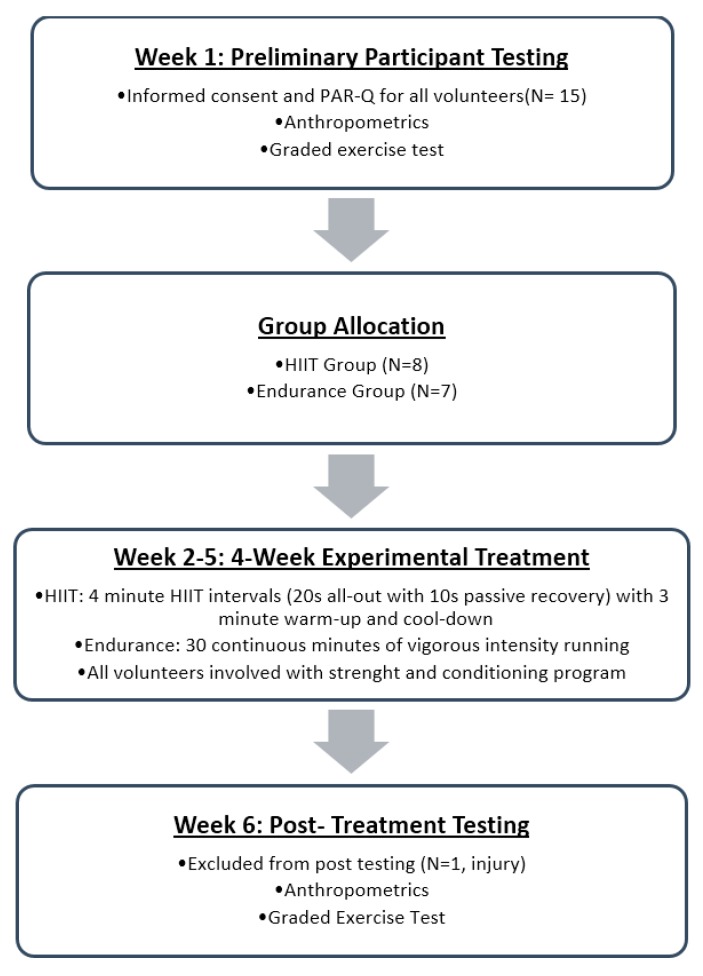
Timeline of experimental design.

A timeline of the study design is shown in Figure 1. Preliminary testing was completed during week one. The four-week training program began at least 24 h following the last participant’s preliminary testing session. Finally, post-training testing was completed at least 24 h after the final training session date during week six.

During the first week of the study, all of the participants reported to the exercise physiology laboratory for preliminary testing. During the initial visit, all of the participants completed an informed consent and underwent anthropometric measurements, including height (m), body mass (kg), and body composition measurements via bioelectrical impedance (BIA) using upper-body bio impedance body fat analyzer (Omron Healthcare Inc., Bannockburn, IL, USA) following standard procedures [19,20]. The participants were then familiarized with the testing equipment and test procedures prior to baseline testing. 

Each participant was fitted with a heart rate monitor chest strap (Polar Electro Inc., Lake Success, NY, USA) to allow for the observation of heart rate throughout the testing session. Heart rates were recorded at the end of each minute throughout the submaximal portion of the test and at the end of each 30-s interval throughout maximal testing. Participants then completed a graded exercise test on a treadmill (Trackmaster TMX425C, Full Vision Inc., Drive Newton, KS, USA) to determine $\dot{V}$O_2_ at three submaximal speeds and peak oxygen uptake ($\dot{V}$O_2peak_). The metabolic system (Ultima CPX Metabolic Stress Testing System, MedGraphics Diagnostics Corporation, St. Paul, MN, USA) was calibrated with a 3.0 L syringe, and the carbon dioxide (CO_2_) and oxygen (O_2_) sensors were calibrated using two known gas percentages before each test. Measures of oxygen consumption ($\dot{V}$O_2_) and respiratory exchange ratio were averaged over the last 30 s of each stage throughout each of the testing protocols [21]. 

### 2.3. Running Economy

The effects of off-season training on submaximal running $\dot{V}$O_2_ was also assessed. All of the participants began preliminary and post-training testing sessions by completing a modified graded exercise test [22]. The graded exercise test (see Table 1) consisted of three submaximal stages at a level grade [23] and at three speeds (2.906, 3.129, and 3.343 m·s^−1^) for three minutes with one minute of passive recovery after each stage. Running economy was calculated for each participant as an average of oxygen consumption ($\dot{V}$O_2_) during steady-state exercise (the last 30 s of each given submaximal speed) and was expressed as milliliters of oxygen consumed per kilogram of body mass per kilometer travelled (mL·kg^−1^·km^−1^). 

### 2.4. Peak Oxygen Uptake

After completion of the final submaximal stage for run economy measurements, each participant was allowed a four-minute recovery period, during which the metabolic mask could be removed and the participants were able to consume water *ad libitum*. Participants remained on the treadmill for a fourth stage to volitional exhaustion. The fourth stage began at the same velocity as the first submaximal stage (2.906 m·s^−1^), and the speed was increased 0.134 m·s^−1^ every 30 s until treadmill speed reached 3.442 m·s^−1^. Once the maximal speed of 3.442 m·s^−1^ was reached, incline was increased 1.0% every 30 s until volitional exhaustion. After the completion of the test, participants were asked to remain on the treadmill for a three-minute cool-down period at an easy walk (1.252 m·s^−1^) at 0% grade. Peak oxygen consumption ($\dot{V}$O_2peak_) was determined by taking an average of the oxygen consumption from the final 30 s of maximal testing and was expressed as milliliters of oxygen consumed per kilogram of body mass per minute (mL·kg^−1^·min^−1^). Peak oxygen uptake was confirmed with standard criteria of volitional termination of the graded exercise test, RER > 1.10, HR_max_, within 10–12 bpm of age predicted HR_max_, and a rating of perceived exertion (RPE) of >17 on Borg’s 6–20 Rating of Perceived Exertion (RPE) scale [24]. 

### 2.5. Training Protocol

All of the participants were briefed on the exercise training protocol after the completion of preliminary testing. Following preliminary testing, participants were matched based on $\dot{V}$O_2peak_ tests, and then assigned to a training group. Each participant was given an instruction sheet based upon their grouping, which outlined their individual training protocol, including their target heart rates. All of the participants were also given a heart rate monitor and corresponding watch (Polar Electro Inc., Lake Success, NY, USA), which were worn during each training session. Heart rate data was recorded every 5 s and individual training session heart rate data were saved and downloaded for analysis. Training sessions for both groups were administered each Monday, Wednesday, and Friday for the four-week period at a 7:00 a.m. Either the researcher or a research assistant oversaw both of these sessions in order to monitor the adherence to the program and compliance to the protocol. Location for HIIT was an exercise room located at the University’s indoor gymnasium. The HIT_run_ program consisted of morning steady-state runs that were completed on a 200-m track at the University’s indoor fieldhouse. 

The aerobic training period consisted of 12 training sessions, to be completed three days per week for four weeks. For HIT_run_, each training session consisted of 30 min of running at an intensity of 75–85% of age-predicted HR_max_. The training sessions for the HIIT group began with a three-minute easy run to warm up, and then followed a modified Tabata protocol [25], which consisted of eight rounds of 20 s bouts of either burpees or squat-tuck jumps, with 10 s of recovery between each bout. The burpee or squat-tuck jump was designated for each session by the researcher and were denoted on each participant’s protocol instruction sheet. The prescribed intensity for the HIIT participants was an “all out” effort, with a target heart rate of 75–85% of age-predicted HR_max_. After the completion of four minutes of intervals, the HIIT group also completed a light three-minute cool-down of jogging and walking, bringing the session duration total to 10 min. 

All of the volunteers (HIT_run_ and HIIT) participated in an undulating periodization strength and conditioning program as part of their off-season training. The undulating periodization model allowed for variations in speed, strength, and volume, and included the rotation of light, moderate, and heavy weights [26]. An undulating periodization program was used to avoid overtraining and optimize recovery [27,28].

Upper and lower-body strength was assessed by one repetition maximum testing (1 RM) in the bench press, squat, and deadlift exercises following recognized guidelines [29]. Athletes performed warm-ups with the bar and then at 50%, 70%, and 85% of estimated 1 RM, with the numbers of repetitions decreasing progressively. Athletes lifted one repetition at 85% before testing a first maximum attempt. Each athlete achieved a 1RM for each lift within four attempts. 

Weekly resistance exercise goals varied in focus so that in each week one lift would focus on power, muscular strength, or muscular hypertrophy (Table 2). Accessory training varied weekly in terms of repetitions completed so that for high repetition days, athletes performed with light weight and for low repetition days athletes performed with heavier weights [30]. Athletes lifted three days per week on alternate days from HIIT or HIT_run_ training. 

### 2.6. Statistical Analyses

Descriptive statistics for participant characteristics and dependent variables are presented as mean ± standard deviation. An a priori power analysis was conducted to determine sufficient sample size. Approximately 10 participants were necessary to have 95% power for detecting a moderate effect (f^2^(V) = 0.2) when employing *α* = 0.05 criterion of significance. A 2 × 2 mixed analysis of variance (ANOVA) for group (HIIT, HIT_run_) by time (pre-training, post-training) was used to examine interactions of the independent variables (training condition: HIIT or HIT_run_) on the dependent variables ($\dot{V}$O_2peak_ and RE) after the four-week training program. The analysis was also used to determine differences in the dependent variables between the HIIT and HIT_run_ groups across the four-week training intervention. An independent samples *t*-test was used to determine significant differences in training heart rates between HIIT and HIT_run_ groups. Statistical analyses were computed using IBM SPSS version 22.0, with an established alpha level of 0.05.

## 3. Results

The aim of the study was to examine the changes in $\dot{V}$O_2peak_ and the improvement in RE following four-week exercise training interventions of either high-intensity intervals or high intensity steady-state endurance sessions with a concurrent strength and conditioning program. Descriptive statistics for participants can be found in Table 3. There were no statistically significant differences in body mass or body composition across the two groups following the four weeks of training.

### 3.1. Running Economy

Run economy was calculated as the oxygen cost of running at three submaximal treadmill speeds. There was homogeneity of covariances, as assessed by Box’s test of equality of covariance matrices (*p* > 0.05). There was no statistically significant group (HIIT vs. HIT) by time (pre-training vs. post-training) interaction on RE, *F*(1, 12) = 3.228, *p* = 0.098, partial η^2^ = 0.212. Thus, the main effects of time and group were explored separately. The main effect of time showed no statistically significant difference in RE at the different time points, *F*(1, 12) = 0.201, *p* = 0.662, partial η^2^ = 0.017. Similarly, the main effect of group showed no statistically significant difference in RE between the HIIT and HIT_run_ groups, *F*(1, 12) = 0.510, *p* = 0.489, partial η^2^ = 0.041 (see Table 4).

### 3.2. Peak Oxygen Consumption 

There was homogeneity of covariances, as assessed by Box’s test of equality of covariance matrices (*p* > 0.05). There was no significant interaction between training group time on $\dot{V}$O_2peak_, *F*(1, 12) = 0.005, *p* = 0.942. The main effect of time showed a statistically significant difference in $\dot{V}$O_2peak_ from pre- to post-testing, *F*(1 ,12) = 12.657, *p* = 0.004, partial η^2^ = 0.041. The main effect of group showed no statistically significant difference in $\dot{V}$O_2peak_ between the intervention groups, *F*(1, 12) = 0.290, *p* = 0.600 partial η^2^ = 0.024. 

The average of maximal heart rates for each training session and the average heart rate for each training session are reported in Table 5. There was no statistically significant difference in mean maximal training heart rates between HIIT, 174.23 ± 4.96 bpm, and HIT, 173.26 ± 3.51 bpm (mean ± standard deviation), training groups *t*(22) = 0.548, *p* = 0.224. There was a statistically significant difference in mean training heart rates for the entire training sessions between HIIT, 163.21 ± 5.81 bpm, and HIT_run_ training groups 158.89 ± 4.29 bpm, *t*(22) = 2.069, *p* = 0.05.

## 4. Discussion

**Figure 2:**
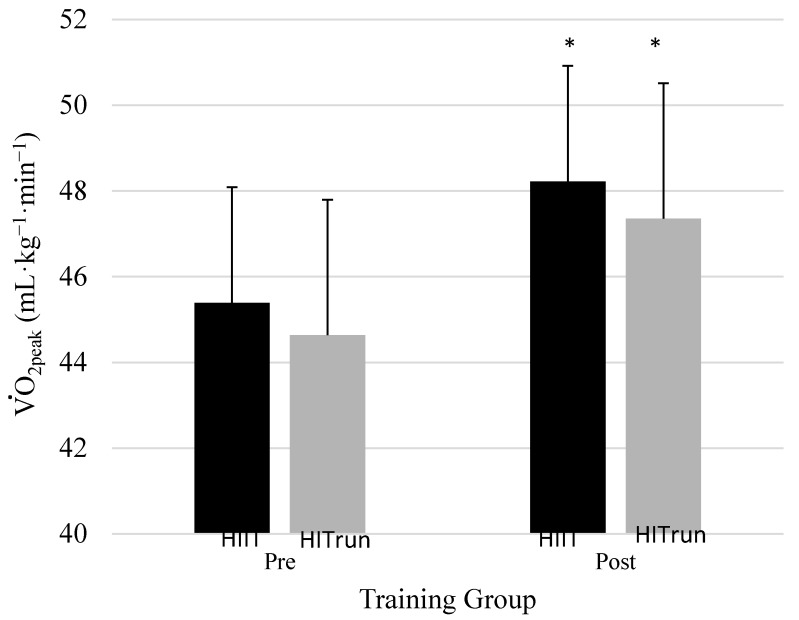
Comparison of changes in $\dot{V}$O_2peak_ from pre- to post-test for both training groups. Post-$\dot{V}$O_2peak_ of HIIT and HIT were significantly different from pre-$\dot{V}$O_2peak_. There were no between-group differences (*p* > 0.05). * *p* < 0.05 significantly different from pre-test of training group, analyzed by 2 × 2 mixed analysis of variance (ANOVA).

Much of the extant literature indicates that HIIT can be a more effective exercise stimulus for improving aerobic capacity than continuous high intensity training, HIT_run_. A major finding of the present study was that aerobic changes ($\dot{V}$O_2peak_) after four weeks of training were significant, regardless of training intervention. The HIIT group increased 6.1% while HIT_run_ group saw an improvement of 6.2%. The extent of the improvement in $\dot{V}$O_2peak_ following the HIIT program is similar to what is reported in the literature. Breil et al. [31] reported a 6% improvement in $\dot{V}$O_2max_ following 15 HIT sessions in junior Alpine skiers. Perry et al. [14] reported a 9% increase in $\dot{V}$O_2max_ following six weeks of HIIT training in recreationally active individuals. Although the HIIT group did not show a significant improvement over the HIT_run_ group, both groups saw an increase in aerobic capacity after the short four-week training intervention (Figure 2). Both groups had similar training intensities, as measured by average maximal training heart rates. The improvements in $\dot{V}$O_2peak_ cannot be solely attributed to the training intervention (HIIT vs. HIT_run_), as all of the participants were also engaged in concurrent strength and conditioning training protocol and two sessions of team practice (see Table 2).

The effect of the short term (four weeks) of HIIT or HIT_run_ training on submaximal $\dot{V}$O_2_ (RE) was also determined. There were no significant differences in RE at all of the submaximal treadmill speeds between groups and pre- and post-training (see Table 4). The short nature of the current training program may not have been significant stimulus to result in changes in RE. Saunders et al. [32] showed a ~4% improvement in RE with highly trained distance runners, yet this was following a nine-week plyometric program. Taipale et al. [33] found a significant increase in RE with runners following eight weeks of concurrent explosive strength and endurance training. It is speculated that potential mechanisms for improved RE with concurrent resistance and endurance training is delayed recruitment of less efficient Type II muscle fibers during steady state submaximal exercise [34]. Although acute bouts of HIIT have been shown to initiate mitochondrial biogenesis signaling pathways [35,36], the current study may not have been significant stimulus to result in changes in RE.

The participants in the study were female field hockey players whose regularly scheduled training coincided with the experimental training. In the first two weeks of the four-week training intervention, the athletes also attended three strength-training sessions per week, each lasting 60 min each. In addition to the HIIT or HIT_run_, and strength training, weeks three and four included three team practice sessions of 60–120 min each. At the end of week three, the team had a scheduled scrimmage day, in which each athlete took part in 150 total minutes of game play. Training volume was calculated as metabolic equivalents (METS) and total training time per week. METS were estimated using pre-training $\dot{V}$O_2peak_ and training intensity, as determined by average training heart rates. HIT_run_ group had almost a three-fold (2.96) greater training volume, yet similar aerobic training benefits as HIIT group. Aerobic training volume and the total weekly exercise time commitment for each of the experimental groups is displayed in Table 6. 

The four-week HIIT training program may not have been a long enough exercise stimulus to see a significant difference between groups, as all of the participants were already highly trained. According to ACSM fitness guidelines, the average $\dot{V}$O_2max_ of both the HIT_run_ and HIIT groups was well within the “excellent” category for aerobic fitness [19]. However, it is important to note that the HIIT group showed significant improvement in $\dot{V}$O_2peak_ from pre- to post-testing and similar improvements in $\dot{V}$O_2peak_ to the HIT_run_ group after the four-week intervention, with a reduced time commitment. A lack of a control group in our study restricts us from definitive conclusions comparing HIIT and HIT_run_, yet the results do support the effectiveness of a short term high intensity training program that is designed to improve aerobic fitness. Although HIIT did not increase aerobic capacity to a greater extent than endurance training, with a shorter time commitment, HIIT was an effective stimulus for improving aerobic conditioning in the female athlete participants in their off-season with less of a time commitment [37]. Additional evidence is needed to generalize the present results to a sedentary population or an athletic male population. 

### Practical Applications

The off-season is important for making improvements in aerobic fitness, strength, and skills without the need to focus on competition. As there are time limitations to the off-season, getting the greatest benefit from each training segment is critical. Given that the aerobic improvements after four weeks of training were significant, regardless of training intervention in this study, it is clear that off- season training is effective. Four weeks of a cardiovascular training plan combined with a strength and conditioning program can improve peak aerobic fitness of these already fit individuals. Student-athletes lead busy lives, trying to balance class schedules with strength training, schoolwork, and practice schedules. Utilizing a HIIT program as a method of conditioning in the off-season or non-traditional season allows for athletes to reap cardiovascular benefits without the extended time commitment of traditional aerobic training. Our results suggest that athletes can obtain similar aerobic benefits in a more time-efficient manner, which could allow for more time to be committed to other important aspects of practice.

## 5. Conclusions

Another notable finding from the study concerns the improvement in peak oxygen consumption relative to the total time necessary to complete each condition. Specifically, each condition evidenced a significant increase in peak oxygen consumption (HIT: 6.2% vs. HIIT: 6.1%). However, the HIIT condition required approximately 20% less time to achieve the improvement than the HIT condition. While future research may explore the mechanisms underlying the improvement, the more immediate implication is that field hockey coaches may want to consider HIIT-type training for their athletes, as doing so may allow for improved aerobic capacity, while simultaneously allowing more time for skill practice and game strategy.

## Figures and Tables

**Table 1:** Graded exercise protocol to determine run economy.

Run Economy Test		
Time (min:s)	Speed	Grade
0:00–2:59	2.906 m·s^−1^	0.0%
3:00–3:59	Rest	-
4:00–6:59	3.129 m·s^−1^	0.0%
7:00–7:59	Rest	-
8:00–10:59	3.353 m·s^−1^	0.0%
11:00–14:59	Rest	-

**Table 2:** Resistance training program for field hockey athletes during non-traditional season.

Resistance Training	Day	Lifts
Main Lift	Day 1	Squat, squat isometric hold to jump, medicine ball side toss
	Day 2	Bench press, squat press, medicine ball throw-down
	Day 3	Deadlift, kettlebell swing, hurdle hop
	Rest Periods	1–3 min depending on focus (power 1–2 min, strength 2–3 min)
Accessory Training	Day 1	*Circuit 1*: Pendlay row, partner bench holds *Circuit 2*: Trap bar deadlift, monster walks *Circuit 3*: Pause thruster, pull up, cable rotations
	Day 2	*Circuit 1*: Dumbbell press, face pulls, landmine row *Circuit 2*: Dumbbell isometric hold, med ball shot put *Circuit 3*: Pause thruster, pull up, cable rotations
	Day 3	*Circuit 1*: Goblet squat, barbell isometric hold *Circuit 2*: Single leg landmine Romanian deadlift, glute bridge *Circuit 3*: Stiff leg pull through, sled back pedal
	Rest Periods	One minute between exercises

**Table 3:** Baseline Descriptive Characteristics of volunteers (N = 14) (Mean ± SD).

Anthropometric Measurements	HIIT (N = 8)	HIT_run_ (N = 6)
Age (y)	19.25 ± 0.89	19.33 ± 1.03
Height (m)	1.63 ± 0.07	1.61 ± 0.13
Body Mass (kg)	62.27 ± 4.83	64.35 ± 6.23
BMI (kg/m^2^)	23.41 ±1.40	25.65 ± 7.15
Body Fat (%)	21.2 ± 2.41	21.9 ± 4.74
Lean Body Mass (kg)	49.06 ± 4.00	50.01 ± 2.11
$\dot{V}$O_2peak_ (mL·kg^−1^·min^−1^)	44.64 ± 3.74	45.39 ± 2.80

**Table 4:** Results from Running Economy and $\dot{V}$O_2peak_ Pre-and Post-Training Tests.

Treadmill Test Type	HIIT (N = 8)		HIT_run_ (N = 6)	
	Pre	Post	Pre	Post
**Running Economy Test**				
Speed 1	211.2 ± 14.9	212.3 ± 10.1	209.7 ± 13.6	214.9 ± 25.4
(2.906 m·s^−1^)				
Speed 2	214.1 ± 16.4	212.2 ± 10.5	213.8 ± 18.1	216.6 ± 21.2
(3.129 m·s^−1^)				
Speed 3	209.6 ± 15.1	211.4 ± 11.4	208.7 ± 16.8	211.2 ± 17.9
(3.353 m·s^−1^)				
$\dot{V}$**O_2peak_ Test**				
$\dot{V}$O_2peak_	44.64 ± 3.74	47.35 ± 3.16 *	45.39 ± 2.80	48.22 ± 2.42 *
Peak RER	1.10 ± 0.05	1.15 ± 0.07	1.12 ± 0.04	1.12 ± 0.06

Note: Running economy: mL·kg^−1^·km^−1^. $\dot{V}$O_2peak_: mL·kg^−1^·min^−1^. RER: Respiratory Exchange Ratio, * *p* < 0.05 significantly different from pre-test of training group, analyzed by 2 × 2 mixed analysis of variance (ANOVA).

**Table 5:** Maximal and average training heart rates for High Intensity Interval Training (HIIT) and High Intensity Training (HIT) programs.

Training Intensity	HIIT (N = 8)		HIT_run_ (N = 6)	
	Max HR	Average HR	Max HR	Average HR
Absolute Training Intensity (bpm)	174.23 ± 4.96	163.21 ± 6.10 *	173.27 ± 3.51	158.90 ± 4.30
Relative Training Intensity (%)	86.79	81.30	86.35	79.18

Note: Relative training intensity calculated as a percent of age predicted HR_max_. * *p* = 0.05 HIIT average HR significantly different from HIT_run_ average HR, analyzed by independent samples *t* test.

**Table 6:** Exercise time commitment for groups (minutes per week) and training volume (METs × minutes per week).

Training Type	Week 1	Week 2	Week 3	Week 4	Total
HIIT	30	30	30	30	120
HIT_run_	90	90	90	90	360
Strength Training					
HIIT	180	180	180	180	720
HIT_run_	180	180	180	180	720
Team Practice					
HIIT	0	0	300	300	300
HIT_run_	0	0	300	300	300
Competition					
HIIT	0	0	150	0	150
HIT_run_	0	0	150	0	150
Weekly Total					
HIIT	210	210	660	510	1590
HIT_run_	270	270	720	570	1830
Weekly Aerobic Total					
HIIT	30	30	480	330	870
HIT_run_	90	90	540	390	1110
Weekly Aerobic Training Volume					
HIIT	330	322	392	320	1364
HIT_run_	1045	1013	991	991	4040
